# Supplementary material for: Efficacy Assessment of Five Policosanol Brands and Damage to Vital Organs in Hyperlipidemic Zebrafish by Six-Week Supplementation: Highlighting the Toxicity of Red Yeast Rice and Safety of Cuban Policosanol (Raydel®)
Source: Pharmaceuticals (Basel). 2024 May 31;17(6):714. doi: 10.3390/ph17060714 (PMC11206962; doi:10.3390/ph17060714)
Supplement: Supplementary file 1 [file pharmaceuticals-17-00714-s001.zip › pharmaceuticals-3037688-supplementary.pdf]

Supplementary Materials

Table S1: Composition of different policosanol brands

Product Code	Product manufacturer name, Country of production	Ingredients (as indicated in label)	Policosanol amount (mg) in tablet
**PCO1**	Raydel-policosanol, Australia	Policosanol-sugar cane wax alcohol, lactose mixed powder, crystal cellulose, hydroxypropylmethyl-cellulose, calcium carboxymethylcellulose, magnesium stearate, the garden blue pigment, titanium dioxide (color), glycerin fatty acid ester milk	20
**PCO2**	Solgar–policosanol, USA	Policosanol, microcystalline cellulose, vegetable cellulose, vegetable magnesium stearate	20
**PCO3**	NutrioneLife-monacosanol, South Korea	Red Yeast Rice powder 5 mg, octacosanol-containing, crystal cellulose, hydroxypropyl-methylcellulose, calcium carboxymethylcellulose, magnesium stearate, silicon dioxide, glycerin	12
**PCO4**	Mothernest–policosanol, Australia	Sugarcane wax alcohol 20 mg, tableting aids	20
**PCO5**	Peter & John–policosanol, New Zealand	Policosanol 33.4 mg (equivalent to octacosanol 20 mg), tableting aids	33.4

Supplementary Material S1:

1. List of the used chemicals: N-ε-carboxylmethyllysine (CAS-No 941689-36-7, Cat#14580-5g), dihydroethidium (DHE, 104821-25-2, Cat #37291), and acridine orange (AO, 65-61-2, Cat#A9231), oil red O (Cat#O0625), and 2-phenoxyethanol (Sigma P1126; St. Louis, MO, USA), paraoxon-ethyl (Cat. No. 36186) and 5-bromo-4-chloro-3-indolyl β D-galactopyranoside (X-gal, Cat#B54252) were procured from Sigma–Aldrich (St. Louis, MO, USA). All other chemicals and reagents else otherwise stated were of analytical grade and used as supplied.

2. Analysis of Plasma

Blood (2 μL) was drawn from the hearts of the adult fish, combined with 3 μL of phosphate-buffered saline (PBS)-ethylenediaminetetraacetic acid (EDTA, final concentration, 1 mM) and then collected in EDTA-treated tubes. The plasma total cholesterol (TC) and triglyceride (TG) were determined using commercial assay kits (cholesterol, T-CHO, and TGs, Cleantech TS-S; Wako Pure Chemical, Osaka, Japan) as per the method suggested by the suppliers.

In brief, 5 μL plasma was mixed with 200 μL reaction mixture (supplied with a commercial assay kit) for the TC analysis. The content was incubated at 37°C for 10 min, resulting in a dark pink-colored product quantified by adsorption at 490 nm (Microplate reader, Bio-Rad, Hercules, CA, USA). Similarly, 5 μL plasma was mixed with a 200 μL TG-specific reaction mixture (supplied with a commercial assay kit) for TG analysis. The content was incubated for 10 min at 37°C, and the formed purple colored product was quantified by taking adsorption at 540 nm. For HDL-C analysis, the plasma

was mixed in an equal ratio with the separation solution (supplied with a commercial assay kit), followed by centrifugation at 3,000 rpm for 10 min. The supernatant (20 μL) was collected and blended with a 200 μL reaction mixture of the TC reagent (supplied with a commercial assay kit). After 10 min incubation at 37°C, the dark pink color intensity corresponding to HDL-C was quantified by taking absorption at 490 nm.

The commercial diagnostic kit (Asan Pharmaceutical, Hwasung, Republic of Korea) was used to quantify aspartate transaminase (AST) and alanine transaminase (ALT) levels in the plasma, following the instructions suggested by the manufacturers. Briefly, 5 μL of plasma was combined with 250 μL of either AST or ALT-specific solution, as supplied in the diagnostic kit. Following a 30 min incubation for AST or 60 min incubation of ALT at 37°C, the mixture was then blended with 250 μL of the respective coloring reagent (AST or ATL-specific, provided in the diagnostic kit). After a subsequent 20 min incubation at RT, 250 μL of 0.4 N NaOH was introduced to halt the reaction. Finally, the AST and ATL were quantified by measuring absorbance at 490 nm.

3. Preliminary determination of toxicity during 3 weeks supplementation

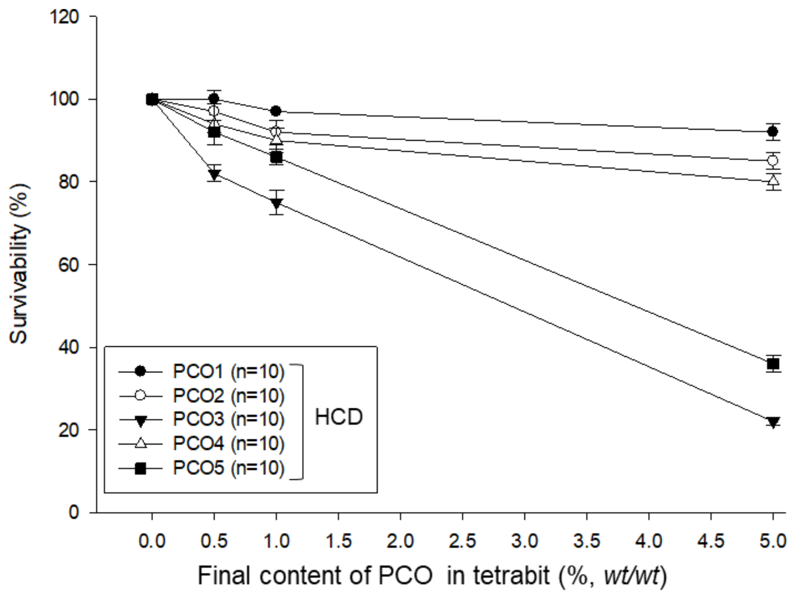

Supplementary Figure S1: Survivability of zebrafish after 3 weeks of consuming various policosanols (PCO1-PCO5) at final concentrations of 0.5%, 1%, and 5% (wt/wt) incorporated into a high-cholesterol diet (HCD). The HCD was prepared by blending cholesterol (final 4%, wt/wt) with a standard zebrafish diet (ND).
